# A systematic review of the clinical effectiveness of acupuncture for allergic rhinitis

**DOI:** 10.1186/1472-6882-8-13

**Published:** 2008-04-22

**Authors:** Jonathan Roberts, Aarnoud Huissoon, Janine Dretzke, Dechao Wang, Christopher Hyde

**Affiliations:** 1 West Midlands Health Technology Assessment Collaboration. Department of Public Health and Epidemiology, University of Birmingham, UK; 2 Department of Immunology, Birmingham Heartlands Hospital, Birmingham, UK

## Abstract

**Background:**

Allergies cause a considerable burden to both sufferers and the National Health Service. There is growing interest in acupuncture as a treatment for a range of conditions. Since acupuncture may modulate the immune system it could be a useful treatment for allergic rhinitis (AR) sufferers. We therefore assessed the evidence for the clinical effectiveness of acupuncture in patients with AR by performing a systematic review of the literature.

**Methods:**

Searches (to 2007) were conducted in all major databases for randomised controlled trials (RCTs) evaluating the clinical effectiveness of acupuncture in the treatment of AR. No limits were placed on language. Studies were included if they compared acupuncture to a sham or inactive acupuncture treatment (placebo) with or without standard care. Meta-analysis was performed where feasible.

**Results:**

Seven relevant RCTs were included after screening and application of inclusion and exclusion criteria. The trials were generally of poor quality as assessed by a modified Jadad scale, with the exception of two studies which scored highly. A wide variety of outcomes was measured but most assessed symptom severity on a visual analogue scale. A meta-analysis failed to show any summary benefits of acupuncture treatment for symptom severity scores or serum IgE measures which could not have been accounted for by chance alone. Acupuncture was not associated with any additional adverse events in the trials.

**Conclusion:**

There is currently insufficient evidence to support or refute the use of acupuncture in patients with AR. A large well conducted RCT, which overcomes identified methodological problems in the existing RCTs, would be required to resolve this question.

## Background

Allergies are responsible for an estimated annual expenditure of £1 billion in the National Health Service (NHS) [1]. Allergic Rhinitis (AR) is a complex of symptoms associated with atopic hypersensitivity reactions to common allergens including house dust mite, animal dander and pollens from grasses, trees and weeds. The prevalence of AR is highest in developed countries. In Western Europe, AR is estimated to affect up to 23% of the population [1-3].

Acupuncture developed from the traditional Chinese medicine techniques that can trace recorded origins back to the 2^nd ^century BC [4]. Acupuncture involves the stimulation of acupoints that are located at lines of meridians that correspond to the flow of energy through the body. Modern acupuncture has evolved other methods of stimulating acupoints including the use of an electrical current, by applying pressure to the acupoint (acupressure) or using a low intensity laser [5,6]. Complementary and alternative medicine (CAM) treatments are becoming increasingly popular in the UK. It is estimated the UK spends an average of £1.6 billion per year on CAM with acupuncture the fourth most common treatment behind aromatherapy, homeopathy and herbal medicines [7].

There is some biological plausibility to the use of acupuncture for allergies. A number of small studies suggest that acupuncture can modulate levels of cytokines and other anti-inflammatory mediators, although the net effect of these changes is not necessarily anti-inflammatory nor would they predictably attenuate allergic disease [8]. Acupuncture can stimulate the release of β endorphin, which, coupled to the release of adrenocorticotrophic hormone (ACTH) [9] acts on the adrenal cortex to stimulate the release of cortisol, offering another possible anti-inflammatory effect [5,8-10].

If it were proven to be effective, acupuncture would be an attractive alternative to conventional symptomatic treatment for some patients. Previous studies have shown that side effects are rare in acupuncture and generally only minor, such as irritation at the needle site [11]. The cost of acupuncture sessions is likely to be comparable to that of symptomatic medication. In addition many patients dislike, and therefore do not adhere to, daily prophylactic medication use, and a drug-free, safe treatment option therefore has considerable attractions. In contrast many health policy makers remain sceptical about acupuncture and reluctant to commission acupuncture services while its effect remains unsubstantiated

The aim of this systematic review is to evaluate the evidence of effectiveness of acupuncture for AR.

## Methods

### Search Strategy

Literature searches were conducted in MEDLINE (1966-August 2007), EMBASE (1988-August 2007), the Cochrane library (including DARE and CENTRAL), the British Library Allied and Complementary Medicines database (AMED 1985-August 2007) and the National Research Register for ongoing trials. The specialist acupuncture library Acubriefs [12] was searched as was the Chinese literature via the database provided by the China National Knowledge Infrastructure (CNKI) website [13]. Index terms for 'acupuncture' 'allergic rhinitis' 'hay fever' and 'clinical trial' were used (see Additional file 1). The bibliographies of included studies were searched for additional references. All studies were collected and organised using the Refman software package (Version 11, Thompson ISI ResearchSoft).

### Study Selection

Randomised controlled trials (RCTs) of acupuncture treatment for patients with AR were included in this review. RCTs were the only study design chosen to minimise any potential for bias where non-objective or patient reported outcomes are used in studies. Inclusion criteria were kept as broad as possible to include subjects with persistent AR (seasonal or perennial) with or without other allergies such as asthma. Patients of any age were included. Any form of acupuncture treatment that stimulates an acupoint was included such as solid needles, electro-acupuncture and laser acupuncture. Sham or fake acupuncture treatment with or without standard care was the comparator. Any outcome measure relating to the effect of treatment was sought including quality of life (QoL), days off work or school, rhinitis symptom scores, medication usage score and adverse effects. Studies published in any language were included.

Non- RCT evidence, studies investigating patients with non-allergic rhinitis or without a placebo (sham or inactive acupuncture) control were excluded.

### Assessment of methodological quality

A modification of the Jadad scoring system [14] was used to asses the quality of the evidence found (Table 1). The scoring system enabled a maximum score of eight for each study and assessed quality parameters such as baseline characteristics, blinding, allocation concealment and drop out rates. A score of 1 was given for each parameter fulfilled, (with an additional point for adequate description of the method) and 0 for those not fulfilled or where this could not be assessed.

**Table 1:** Quality Assessment of included studies.

Study	Randomisation (appropriate description?)	Concealment (appropriate description?)	Patient blinding (appropriate description?)	Withdrawals and dropouts	Comparable baseline groups	Total (max 8)
Wolkenstein 1996 [16] & Horak 1993 [18] 1998 [17]	1	0	0	0*	0	2
Langer & Hauswald 1989 [19]	1	0	0	1	0*	2
Ng et al 2004 [21]	1 (1)	1 (0)	1 (1)	1	1	7
Magnusson et al 2004 [15]	1 (1)	0	1 (1)	1	0	5
Williamson et al 1996 [23]	1 (1)	1 (1)	1 (1)	1	0*	7
Petti et al 2002 [20]	1 (0)	0	0	1	1	3
Xue et al 2002 [22]	1 (0)	0	1 (1)	1	0	4

* Information unclear

A score of 1 was given for each parameter fulfilled in the text with an additional point for adequate description of randomisation, allocation concealment and blinding. A 0 was scored where no description or detail was given.

### Data extraction and statistical methods

Data from included studies was extracted using a structured pro-forma (see Additional file 2). Where possible the results from studies were summarised quantitatively using the Revman software package (Version 4.2, Cochrane Collaboration, Oxford). Data of interest were continuous measures, so the relevant effects were differences between the means, summarised as weighted mean differences or where different scales were used to measure the same general attribute, standardised mean differences. Heterogeneity was assessed using the chi^2 ^test and I^2 ^test for heterogeneity. A random effects model was preferred where there was marked heterogeneity (I^2 ^> 50%) and a fixed effects model in other circumstances. A funnel plot was the intended method of detecting small study and publication bias if sufficient included studies had been detected.

Quality assessment and data extraction were completed independently by two reviewers for English language studies and singly for non-English language studies. Any discrepancies were discussed and resolved between reviewers, but inter-rater reliability was not formally assessed.

## Results

**Figure 1:**
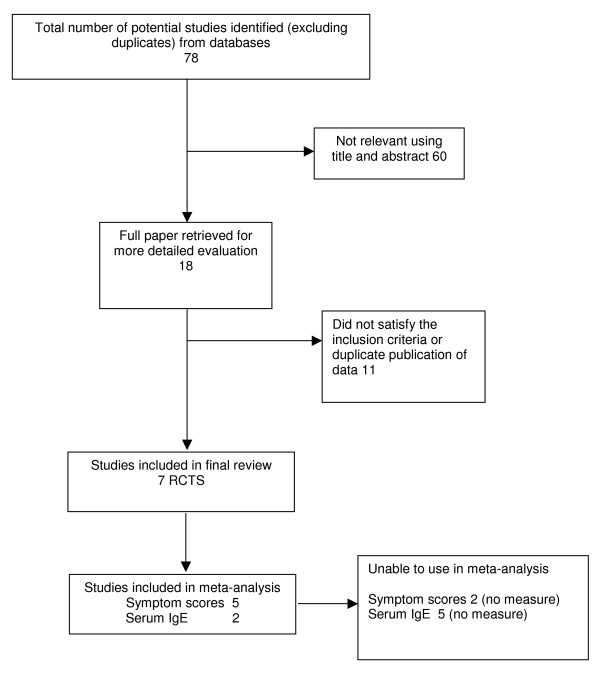
Flow of included studies.

Seven trials met the inclusion criteria (details of study flow are given in Figure 1). Five trials were published in English language, two were published in German. Of the excluded studies two were in English, two in German, two in Chinese and one in Czech (see Additional file 3).

The majority of trials were conventional RCT's, one trial was conducted in an experimental setting using a chamber with a regulated flow of pollen. One trial adopted a cross-over RCT design (full details of included studies are in Table 2).

**Table 2:** Details of included studies

**Author, year, place of study**	**Population**	**Intervention/Comparator**	**Outcomes**	**Result summary**
Wolkenstein 1996 [16] & Horak 1993 [18] 1998 [17] Germany	18–65 yrs with AR for > 2 yrs n = 30 randomised (unclear on numbers in each group). Mean age 41 yrs.	Needle acupuncture Placebo needle was placed away from defined acupoint. 1 session per week, total 9 sessions.	1) Nasal secretions (30 mins) 2) Subjective well-being VAS (15 mins) 3) Subjective symptom score (15 mins) 4) FEV1 (30 mins) 5) Change in the nasal flow (15 mins) 6) Daily symptom diary (two months)	1) + 5)There was no difference in the sum of nasal flows between the groups 2) There was no difference in subjective well being 3) + 6) No difference for symptom score measures or the daily symptom diary scores 4) No difference reported
Langer & Hauswald 1989 [19] Germany	Needle Intervention group n = 22 mean age 33 Laser intervention n = 26 no further details Control group n = 17 no further details	Needle acupuncture: 3 sessions per week total 9 sessions. Laser acupuncture: 15 sessions, 5 times per week, 3 weeks Control: as laser but with laser inactive.	1) Nasal swelling 2) Flow of watery secretions 3) Conjunctivitis 4) Medication use 5) Sneezing 6) Subjective patient report of breathing through the nose 7) Patient judgment	Placebo, acupuncture and laser acupuncture significantly reduced nasal swelling, flow of watery secretions and conjunctivitis at the 2% significance level No results were reported for subjective patient measure of breathing through the nose or sneezing.
Ng et al 2004 [21] Hong Kong	> 6 yrs old with symptoms for > 4 weeks and other allergies at two sites. Intervention group n = 35 ave age 11.7 Control group n = 37 ave age 11	Needle acupuncture Control needling at less depth 2 sessions per week for 8 weeks (16 sessions) Follow-up 12 weeks	1) Daily rhinitis score 2) Symptom free days 3) Daily relief medication score 4) Blood eosinophil count 5) Serum IgE levels 6) Adverse events/preference	1) significant reduction in daily symptom score in the intervention group 2) An increase in symptom free days in the intervention group from baseline reported 3–6) There was no change in symptom relief scores between the groups or the serum markers measured.
Magnusson et al 2004 [15] Sweden	18–50 yrs old Intervention n = 20 ave age 35.3 Control n = 20 ave age 35.3	Needle acupuncture Control acupuncture away from intervention acupoints and shallow needling 12 sessions of 3 needling events in 30 mins. Follow-up 12 months	1) Allergic symptoms (VAS) 2) Mediation use 3) Allergic symptom due to pollen (VAS) 4) Tiredness during pollen season 5) Depression during pollen season 6) Impaired ability to work (VAS) 7) Serum IgE	1–6) no significant findings between intervention and control groups for any of the outcome measures. 7) Serum IgE levels were found to be reduced in the intervention group for the mugwort allergen, but there were differences in baseline characteristics between the groups.
Williamson et al 1996 [23] UK	> 16 yrs with moderate or severe symptoms Intervention n = 51 ave age 31.9 Control n = 51 ave age 29.9	Needle acupuncture Control group – needling on the patella 5 minute sessions 3/4 times per week	1) Patients in remission 2) Mean weekly symptom score 3) Units of medication 4) Perceived effect of acupuncture	1)+2) no changes in remission of symptoms over the course of the study or weekly symptom score 3) There was no difference between medication use score 4) There was no difference between perceived effect
Petti et al 2002 [20] Italy	22–45 yrs with > 2 yrs symptoms Group A: Plasma reference control. Healthy individuals n = 30 22–42 yrs Group B1: Intervention. n = 30 22–45 yrs Group B2: Sham Intervention n = 30 24–45 yrs Group B3: patient control no intervention. n = 30 age not clear	Needle acupuncture with electrostimulation of the needle once in place. In the intervention group the needles in points ST36 and LI4 were electrostimulated with pulsating waves for 15 mins. All sessions lasted ~20 mins	1) Symptom score (5-point scoring system) 2) Serum cytokine measurements for IL-2, IL-6 and IL-10 Measures were taken 2 hrs and 24 hrs after treatment	1) Groups B1 and B2 showed an improvement in symptom scores after acupuncture despite B2 receiving sham acupuncture. 2) Serum IL-2 cytokine levels were increased after 24 hours in the reference control group and group B1. However all values were still within the normal range Serum IL-6 levels did not change in any groups over the 24 hrs. Serum IL-10 levels were significantly reduced in the active intervention group
Xue et al 2002 [22] Australia	Age range 18–70 years > 2 years duration Group A: n = 17 subjects, ave age 44 Group B: n = 13 subjects, ave age 44	Needle acupuncture administered 3 times a week for 4 weeks, sessions lasted 25 minutes. Sham group received shorter needles.	1) Severity symptom score 2) Relief medication score 3) Side effects	1) There was a significant improvement in post treatment symptom severity scores between intervention and control groups 2) One patient in each group required relief medication – no analysis was performed. 3) no side effects reported

Participants in the studies were recruited either via advertisements in GP practices or in the local media. This volunteer population may not be a true representation of the general AR suffering population. The participants in trials were generally adults with the exception of one study which was set in a paediatric respiratory clinic but included some adults

The diagnosis of AR was variable: Two studies included patients with perennial rhinitis, while six studied exclusively patients with seasonal symptoms. Details of diagnosis were poorly reported, with no details given in some studies and others simply confirming that patients had positive tests for allergies (skin prick tests or specific IgE measurement) without further details.

The intervention varied considerably across the trials. One study used pre-seasonal treatment, whereas the others treated patients when symptomatic. The frequency of treatment sessions ranged from one to five per week with a total between two and 16 sessions overall. One trial evaluated the use of laser acupuncture compared to needle acupuncture, but also included a no treatment group allowing comparison between acupuncture and a control group. The acupoints selected by practitioners were detailed in five of the studies. There were 24 different acupoints used in these trials. A wide range of outcome measures was used, with the most common being symptom severity score measured on a visual analogue scale (VAS) and medication use.

The comparator in trials was a placebo acupuncture treatment that generally involved needling away from the active site or needling at a more shallow depth with shorter needles (to avoid stimulating the acupoint).

### Trial quality

Only three studies achieved quality scores of greater than four from a maximum of score of eight (Table 1). All of the trials were described as randomised but only three of the seven trials gave details of the method used. Only two of the trials included information on allocation concealment. Due to the nature of the intervention it is difficult to tell if the intervention was distinguishable from the control treatment or not. Blinding of patients and/or outcome assessors was mentioned in four of the trials. An intention to treat (ITT) analysis was stated in only one trial, but even here it was not possible to assess if this had really been carried out. Losses to follow-up were described in all trials and were minimal. In three reports the baseline characteristics of the study groups were not comparable, and in two further studies comparability could not be adequately assessed from the details given. This may be due to the low numbers of participants in some trials, but raises quality concerns. Only one study addressed the issue of sample size and power.

### Symptom severity scores

Five trials reported symptom severity in a way that could be used in meta-analysis, and in one further study the authors kindly provided the raw data for meta-analysis [15]. The study by Wolkenstein et al [16-18] could not be incorporated as it reported baseline VAS scores with means and median values, but only reported the outcomes as medians. One study did not report symptom severity scores [19] but investigated percentage change in symptoms from a pre-season level set to 100%. This study reported a significant change (at the 2% level) in nose swelling and conjunctivitis after treatment for both acupuncture and placebo groups, but did not report a comparison between the groups.

**Figure 2:**
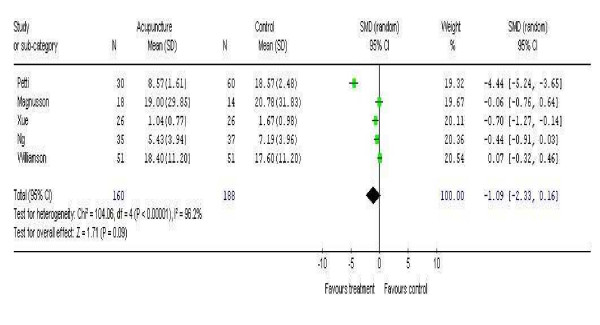
Meta-analysis of five studies investigating a change in symptom severity score.

There was a high degree of heterogeneity between the studies when combined in the meta-analysis (Chi^2 ^104.06 p = 0.00001 I^2 ^96.2%) with a total of 160 subjects in the intervention arm and 188 in the control arm, which was associated with a non-significant trend in favour of the intervention, -1.09 (95%CI -2.33 to 0.10) (Figure 2). Two of the studies have shown a positive treatment effect in contrast to the other studies and the results are dominated by one study that has shown a strong treatment effect in favour of the intervention [20]. This study however was of uncertain quality; when this study was excluded as a sensitivity analysis, the degree of heterogeneity dropped (Chi^2 ^16.77 p = 0.002 I^2 ^76.1%) leaving 172 patients in the intervention arm and 169 in the control arm. The overall effect estimate continued to show a non-significant trend in favour of the treatment of -0.43 (95% CI -0.89 to 0.02).

### Clinical significance of improved symptom severity

It is difficult to interpret the clinical significance of any magnitude of change in symptom score on a VAS. One trial correlated the improvement in daily rhinitis score with a significant increase in the number of symptom free days (from baseline 3.2 to 12.7 after follow-up in the acupuncture group compared to baseline 1.38 to 2.4 in the control group, p = 0.0001) [21]. The pre-treatment baseline outcome values of the groups in this trial, although similar for rhinitis score, were different for symptom free days and this might confound this conclusion [21].

The results of the Xue et al [22] study were also broken down to changes in nasal and non-nasal symptoms, which were both significant. However, this cross-over trial did not have a wash out phase between the cross-over of treatments. This design also does not take account of variable pollen counts and resultant symptom severities that may occur during the course of a pollen season. The authors did not comment on possible carry-over of treatment effect between phases.

### Serum and plasma biomarkers

**Figure 3:**
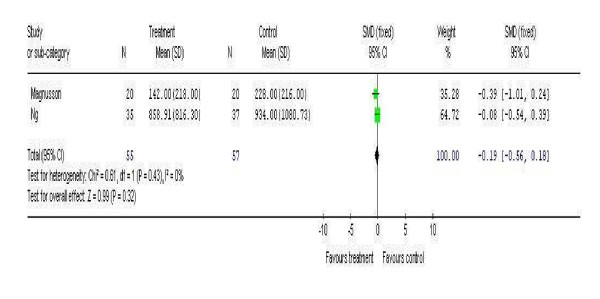
Meta-analysis of two studies investigating changes in serum IgE levels.

Two studies included measurement of serum IgE levels [15,21]. The studies were combined in a meta-analysis (Figure 3) and included a total of 55 patients in the intervention arm and 57 in the control arm. There was very little heterogeneity between these studies (Chi^2 ^0.61 p = 0.32 I^2 ^0%) and the effect estimate was modestly in favour of the intervention (-0.19) but was not significant (95% CI -0.56 to 0.18).

One study investigated plasma cytokine levels and found a reduction in plasma IL-10 levels 24 hours after treatment in the active acupuncture arm [20]. Plasma IL-10 levels were shown to be significantly reduced in the intervention group from baseline 138.1 +/- 28.5 ng/ml to 74.9 +/- 21.4 ng/ml (p < 0.05).

### Changes in Medication use

One trial reports a significant reduction in drug usage in the acupuncture group [19]. Pre-season use was set at 100% and in the acupuncture group medication use fell from 550% to 75% over the pollen season compared to the placebo group (263% to 88%). Magnusson et al [15] showed no differences between intervention and control groups in medication use. Williamson et al [23] showed no difference in the unit use of medication between groups.

### Adverse events reported in trials

None of the studies reported any severe adverse events associated with the use of acupuncture.

## Discussion

This is, to our knowledge, the first comprehensive review that has specifically investigated the use of acupuncture in AR that has followed the standards guidelines of the Quality of Reporting of Meta-analyses (QUOROM) recommendations for the reporting of systematic reviews and meta-analysis [24]. There were no restrictions applied to language and a number of literature databases were searched using a comprehensive search strategy. It was not possible to combine the results of all the trials due to the variety of outcome measures used. Data was obtained from authors where possible allowing pooling of some results into a meta-analysis which has not previously been published in this field. We cannot assess publication bias statistically in such a small collection of studies.

The quality scores of the studies were generally poor, although two scored very highly. While some of the studies pre-date the CONSORT guidelines for reporting RCTs, omission of such quality data from later studies is a serious concern. We nonetheless included these in the analysis since they fulfilled our inclusion criteria. Given the difficulty of performing RCTs in acupuncture all available data should contribute to a final analysis.

The meta-analysis of AR symptom scores showed no overall effect of acupuncture. This conclusion may be confounded by the generally low patient numbers and variable reporting of outcomes. Inclusion of Wolkenstein's data would have increased the number of patients analysed, but this was not available in a form that could be included in our meta-analysis. While there is a trend in favour of a positive effect of acupuncture, this is largely attributable to one study which scored poorly on quality assessment (19). The higher quality studies tended to show lower overall effects.

Our inclusion criteria stipulated that studies had to compare acupuncture with a control sham or inactive acupuncture (placebo intervention). Interestingly one of the excluded studies [25] compared acupuncture with standard care alone. Outcomes measured in this study included symptom severity and serum IgE, with IL-4 and Interferon gamma (IFNγ) levels. There was a significant improvement in long term cumulative score of symptom before and after treatment and a significant decrease of IgE and IL-4 for the acupuncture and standard care groups, but this did not reach significance between groups. The effects of acupuncture may be more marked when compared to a non-blinded comparator, highlighting the importance of sham control in acupuncture studies [26].

Other outcome measures such as drug use or QoL were either poorly reported or not reported across the studies. Likewise, the measurement of serum and plasma biomarkers such as IgE and cytokines was performed in only a few studies. While some individual studies have reported significant changes, the biological significance of these observations is questionable. The report of active acupuncture reducing serum levels of IL-10 contradicts evidence from allergen-specific immunotherapy, where active treatment is associated with an increase in IL-10 production [27]. Overall these measures have not contributed usefully to the summative assessment of the evidence for acupuncture in AR. Any future use of such indices in trials of acupuncture in AR should focus on those which have been shown to be helpful in trials of proven therapeutic modalities such as allergen-specific immunotherapy.

A previously published systematic review investigating a number of CAM treatments for rhinitis and asthma was identified [28]. The review was published by the international board of the Allergic Rhinitis and its Impact on Asthma (ARIA) collaboration in 2006 [28]. It included studies on acupuncture, herbal medicines, homeopathy and physical techniques (including yoga, chiropractic and educational programs). This large review has attempted to answer a very broad question. It is limited in the fact that it only included studies published in the English language and the searches were only conducted in MEDLINE and the Cochrane library [28]. The review contained four trials relevant to acupuncture in rhinitis, three of which are included in this review (one study included patients with non-allergic rhinitis so was not included in this review). The authors note the few RCT data available for AR and that the majority of studies outside of RCTs are 'not randomised, controlled or descriptive' [28]. By focussing specifically on acupuncture for AR and using comprehensive search strategies across all publication languages we have been able to add to this evidence base by including four additional RCTs not identified in this previous review, and combine the results in a meta-analysis. The results of our review further confirm the previous work and strengthen the conclusion that there is currently insufficient high quality evidence to support or refute the use of acupuncture for AR [28].

## Conclusion

It is not possible to recommend acupuncture as a proven treatment for AR on the basis of published evidence. However this evidence is derived from inadequate clinical trials. Studies in AR frequently demonstrate marked improvements in the placebo group, and this means that large, well-controlled studies are required to demonstrate true effects. The quality of future trials could be improved by utilizing the proposed standardised reporting of acupuncture studies, similar to the CONSORT statement [26,29,30] and use of standardised outcome measures for conventional studies in AR [30]. Given the high prevalence of AR, and the popularity of CAMs such as acupuncture, recruitment to such a study should be limited only by the availability of suitable study centres with the required expertise in both allergy and acupuncture.

## Competing interests

The authors declare that they have no competing interests.

## Authors' contributions

JR performed the literature search, applied inclusion criteria, data extracted, performed the meta-analysis and wrote the first draft of the manuscript. AH applied inclusion criteria, data extracted and edited the manuscript. JD translated German studies, applied inclusion criteria, data extracted and edited the manuscript. DW translated Chinese studies, applied inclusion criteria, gave statistical advice and edited the manuscript. CH gave methodological advice, participated in the design of the review and edited the manuscript. All authors read and approved the final manuscript.

## Author's note

After completing this review the following article was published: Xue CC et al Acupuncture for persistent allergic rhinitis: a randomised, sham-controlled trial. MJA 187 (6): 337-341, 2007[31]

This well conducted trial evaluated the use of acupuncture vrs a sham control acupuncture in patients with persistent AR over an eight week treatment program and 12 week follow-up period. The authors randomised 42 patients to active acupuncture and 38 to a sham control group.

The result of this trial was an improvement in cumulative seven day symptom score (sum of individual scores for nasal obstruction, sneezing, rhinorrhoea and nasal itching) between the active and sham acupuncture groups after treatment (effect size -1.85 in favour of treatment p = 0.01) and at follow-up (effect size -1.87 in favour of treatment p = 0.001) this however did not correlate with any difference in the secondary outcome measure which was relief medication use.

This trial follows the trend in results highlighted in the previously published literature with a moderate improvement in symptom severity score which is difficult to interpret in regard to clear clinical benefit. This therefore does not change the overall conclusions of this review but is highlighted here in the interest of including the most up to date information available.

## Pre-publication history

The pre-publication history for this paper can be accessed here:



## Supplementary Material

Additional file 1 Search Strategy. Basic search strategy and terms used. This was adapted depending on the database being searched. Click here for file

Additional file 2 Data extraction form. Details of data collected from each included study. Click here for file

Additional file 3 Included and excluded studies. Details of included and excluded studies reason for exclusion and references. Click here for file
