# Supplementary material for: A systematic review of the clinical effectiveness of acupuncture for allergic rhinitis
Source: BMC Complement Altern Med. 2008 Apr 22;8:13. doi: 10.1186/1472-6882-8-13 (PMC2386775; doi:10.1186/1472-6882-8-13)
Supplement: Additional file 1 — Search Strategy. Basic search strategy and terms used. This was adapted depending on the database being searched. [file 1472-6882-8-13-S1.doc]

**Additional file 1.**

**Example of search strategy used for Medline search**

1 exp Rhinitis/

2 exp Hypersensitivity/

3 allerg$.tw.

4 hay fever.tw.

5 or/1-4

6 exp Acupuncture/

7 exp Acupuncture therapy/

8 Acupuncture.tw.

9 electroacupuncture.tw.

10 or/6-9

11 Randomi?ed controlled trial$

12 exp Clinical Trial/

13 or/11-12

14 5 AND 10

15 5 AND 10 AND 13
