# Supplementary material for: A systematic review of the clinical effectiveness of acupuncture for allergic rhinitis
Source: BMC Complement Altern Med. 2008 Apr 22;8:13. doi: 10.1186/1472-6882-8-13 (PMC2386775; doi:10.1186/1472-6882-8-13)
Supplement: Additional file 2 — Data extraction form. Details of data collected from each included study. [file 1472-6882-8-13-S2.doc]

**Additional file 2.**

**Data Collected**

Title of Study:

Reference Manager ID Number

full paper/abstract/other:

1st Author/Year/Country:

Data extracted by:

**Population Characteristics**

Sample source:

Setting:(e.g. private clinic, GP, NHS clinic):

In-/Exclusion criteria for patients:

How was allergy confirmed (reference test?):

Total number of patients eligible/recruited:

Number randomised:

Patient characteristics:

**Intervention Control**

n= n=

age age

sex (m/f) sex (m/f)

Allergy history: Allergy history:

Co-morbidity: Co-morbidity

Medication: Medication:

Other/comments: Other/comments:

**Intervention**

Type of acupuncture under investigation:

Person performing acupuncture:

Was the acupoint(s) detailed?

Duration/Frequency of acupuncture:

What is the comparator?

**Outcomes**

State all outcome measures:

How were outcomes assessed (e.g. symptom scores, Serum IgE) and by

whom?

Results: if applicable state individual patient data and/or summary measures with SD/SE/CI, statistical significance (p-value)

**Outcome measure Intervention**

Losses to follow-up:

State losses to follow up for intervention and control groups:
