# Supplementary material for: A systematic review of the clinical effectiveness of acupuncture for allergic rhinitis
Source: BMC Complement Altern Med. 2008 Apr 22;8:13. doi: 10.1186/1472-6882-8-13 (PMC2386775; doi:10.1186/1472-6882-8-13)
Supplement: Additional file 3 — Included and excluded studies. Details of included and excluded studies reason for exclusion and references. [file 1472-6882-8-13-S3.doc]

**Additional file 3. Included and excluded studies**

| **Study** | **Do the patients or study population have allergic rhinitis?** | **Is an Acupuncture treatment compared to a control (inactive or sham)?** | **Are patients randomised to their treatment?** | **Is a relevant outcome measured**  **- symptom scores/quality of life/serum IgE etc** | **Include?** |
| --- | --- | --- | --- | --- | --- |
| Brinkhaus 2004 Allergy[1]  Brinkhaus 2005 Chinesische Medizin[2] | √ | X all patients also given Chinese herbal remedy | √ | Unable to separate herbal treatment and acupuncture | X |
| Chari 1988  Am J Acu [3] | √ | √ | ? not mentioned/  unclear | √ | X |
| Chuanjie 1990  Chinese J Acu Med [4] | √ | X Cohort without control group | X | ? | X |
| Drasnar & Palecek 1981  Cesk Otolaryngol[5] | √ | X Uncontrolled case series | X | √ | X |
| Langer & Hauswald 1989  Dtsch Zschr Akup [6] | √ | √ | √ | √ | √ |
| Magnusson 2004  Am J Chin Med [7] | √ | √ | √ | √ | √ |
| Ng 2004  Pediatrics [8] | √ | √ | √ | √ | √ |
| Petti 2002  J Trad Chinese Med [9] | √ | √ | √ | √ | √ |
| Querfurt 1994  Dtsch.Zschr.Akup [10] | √ | X Uncontrolled before and after study | X | √ | X |
| Rao & Han 2006  Zhongguo [11] | √ | X vs. standard care only | √ | √ | X |
| Williamson 1996  Acupuncture in Medicine [12] | √ | √ | √ | √ | √ |
| Wolkenstein & Horak 1993/1996/1998 [13-15] | √ | √ | √ | √ | √ |
| Xue 2002  Am J Chin Med[16] | √ | √ Cross-over trial | √ | √ | √ |
| Xue 2003  Hong Kong Med J[17] | √ | X all patients given acupuncture Chinese herb being investigated | N/A | Efficacy of herb not acupuncture being tested | X |
| Zhao & Wang 2005  Shaanxi Chinese Medicine  [18] | √ | X | unclear | √ | X |

Reference List

1. Brinkhaus B, Hummelsberger J, Kohnen R, Seufert J, Hempen CH, Leonhardy H, Nogel R, Joos S, Hahn E, Schuppan D: **Acupuncture and Chinese herbal medicine in the treatment of patients with seasonal allergic rhinitis: a randomized-controlled clinical trial.** *Allergy* 2004, **59:**953-960.

2. Brinkhaus B, Hummelsberger J, Kohnen R, Seufert J, Hempen CH, Leonhardy H, et al: **Die behandlung der saisonalen allergischen rhinitis
mit akupunktur und chinesischen arzneimitteln: Ergebnisse einer
randomisierten studie und diskussion klinischer erfahrungen.** *Chinesische Medizin* 2005, **20:**47-58.

3. Chari P, Biwas S, Mann SB, Sehgal S, Mehra YN: **Acupuncture therapy in allergic rhinitis.** *American Journal of Acupuncture* 1988,143-148.

4. Chuanjie L, Yamashiro, Yamabuki T, Kasamatsu, Nishibayashi: **33 cases of allergic rhinitis treated by acupuncture.**  *The Chinese Journal of Acupuncture & Moxibustion* 1990, **3:**185-186.

5. Drasnar T, Palecek D: **Classical acupuncture in the treatment of rhinitis vasomotorica and rhinitis pollinosa.** *Cesk Otolaryngol* 1981, **30:**104-106.

6. Langer H, Hauswald B: **Die therapeutische Wirkung der Akupunktur und
Laserpunktur bei Patienten mit Rhinopathia pollinosa.** *Dtsch Zschr Akup* 1989, **32:**109-111.

7. Magnusson AL, Svensson RE, Leirvik C, Gunnarsson RK: **The effect of acupuncture on allergic rhinitis: a randomized controlled clinical trial.** *Am J Chin Med* 2004, **32:**105-115.

8. Ng DK, Chow PY, Ming SP, Hong SH, Lau S, Tse D, Kwong WK, Wong MF, Wong WH, Fu YM et al.: **A double-blind, randomized, placebo-controlled trial of acupuncture for the treatment of childhood persistent allergic rhinitis.** *Pediatrics* 2004, **114:**1242-1247.

9. Petti FB, Liguori A, Ippoliti F: **Study on cytokines IL-2, IL-6, IL-10 in patients of chronic allergic rhinitis treated with acupuncture.** *J Tradit Chin Med* 2002, **22:**104-111.

10. Querfurt H: **Erfolge und langzeitwirkung der korperakupunktur bei
patientn miy rhinopathia pollinosa und bronchialer betiligung.** *DtschZschr Akup* 1994, **37:**83-87.

11. Rao YQ, Han NY: **Therapeutic effect of acupuncture on allergic rhinitis and its effects on immunologic function.** *Zhonggou Zhen Jiu* 2006, **26:**557-560.

12. Williamson L, Yudkin P, Livingstone R, Prasad K, Fuller A, Lawreance M: **Hay Fever treatment in General Practice: A randomised controlled trial comparing standardised Western acupuncture with sham acupuncture.** *Acupuncture in Medicine* 1996, **14:**6-10.

13. Wolkenstein E: **Acupuncture and allergiology. Was leistet die akupunktur in der allergologie?** *Deutsche Zeitschrift fur Akupunktur* 1996, **39:**124-126.

14. Wolkenstein E, Horak F: **Protective effect of acupuncture on allergen provoked rhinitis.** *Wien Med Wochenschr* 1998, **148:**450-453.

15. Wolkenstein E, Horak F: **Protekitiver Effekt von Akupunktur gegenuber einer mittels Allergen-provokation induzierten rhinitis.** *Dtsch Zschr Akup* 1993, **36:**132-137.

16. Xue CC, English R, Zhang JJ, Da CC, Li CG: **Effect of acupuncture in the treatment of seasonal allergic rhinitis: a randomized controlled clinical trial.** *Am J Chin Med* 2002, **30:**1-11.

17. Xue CC, Thien FC, Zhang JJ, Yang W, Da CC, Li CG: **Effect of adding a Chinese herbal preparation to acupuncture for seasonal allergic rhinitis: randomised double-blind controlled trial.** *Hong Kong Med J* 2003, **9:**427-434.

18. Zhao HY, Wang Y: **Treatment of allergic rhinitis using acupuncture with Guizhi Pingfeng soup.** *Shaanxi Chinese Medicine* 2005, **26**.
